# Microenvironment Cell Contribution to Lymphoma Immunity

**DOI:** 10.3389/fonc.2018.00288

**Published:** 2018-07-27

**Authors:** Deepika Kumar, Mina L. Xu

**Affiliations:** Departments of Pathology & Laboratory Medicine, Yale University School of Medicine, New Haven, CT, United States

**Keywords:** lymphoma, microenvironment, T cell subsets, stromal cells, lymphoma exosomes

## Abstract

Lymphoma microenvironment is a complex system composed of stromal cells, blood vessels, immune cells as well as extracellular matrix, cytokines, exosomes, and chemokines. In this review, we describe the function, localization, and interactions between various cellular components. We also summarize their contribution to lymphoma immunity in the era of immunotherapy. Publications were identified from searching Pubmed. Primary literature was carefully evaluated for replicability before incorporating into the review. We describe the roles of mesenchymal stem/stromal cells (MSCs), lymphoma-associated macrophages (LAMs), dendritic cells, cytotoxic T cells, PD-1 expressing CD4+ tumor infiltrating lymphocytes (TILs), T-cells expressing markers of exhaustion such as TIM-3 and LAG-3, regulatory T cells, and natural killer cells. While it is not in itself a cell, we also include a brief overview of the lymphoma exosome and how it contributes to anti-tumor effect as well as immune dysfunction. Understanding the cellular players that comprise the lymphoma microenvironment is critical to developing novel therapeutics that can help block the signals for immune escape and promote tumor surveillance. It may also be the key to understanding mechanisms of resistance to immune checkpoint blockade and immune-related adverse events due to certain types of immunotherapy.

## Background

The cellular context in which lymphoma cells thrive has only recently become an important focus of inquiry. The roles of what used to be considered passive bystanders are quickly becoming elucidated in order to parse out potential targets for immunotherapy. Although our understanding of cytogenetic abnormalities and molecular pathways in lymphoma are in advance of solid organ tumors, the same cannot be said of the tumor microenvironment. In this section, we summarize some of the major components of the lymphoma microenvironment and their contribution to lymphoma immunity.

The primary goal of this review is to address the interplay between lymphoma cells and the cells of the lymphoma microenvironment and to understand how this communication leads to mechanisms of immune evasion and tumor proliferation. Our manuscript will also present some of the controversies in the field and present the limitations in our understanding of the roles and responsibilities of the microenvironment cell in lymphoma pathogenesis.

## Introduction

Lymphomas are a diverse group of clonal neoplasms arising from B and T lymphocytes, and natural killer (NK) cells and are characterized by infiltration of lymphoid structures. Most of these neoplasms correspond to the normal stages of B-cell or T-cell differentiation and hence can be classified accordingly (1). Advances in structural and functional genomics have highlighted the underlying genetic aberrations and oncogenic regulatory pathways leading to a better understanding of the molecular pathogenesis of lymphomas (2). In contrast, the integral role played by microenvironment in lymphomagenesis and progression has only been recently highlighted and needs to be explored in greater depth.

## Lymphoma microenvironment

In addition to somatic mutations and inflammation, the role of tumor microenvironment (TME) in acquisition of key characteristics of cancer pathogenesis and progression, like sustained tumor proliferative signaling, resisting cell death, evasion of growth suppressors, and immune escape mechanism is becoming important in the study of lymphoma pathogenesis (3). The lymphoma microenvironment is increasingly being recognized as a dynamic and interactive supporting network of immune cells, stromal cells, cytokines, blood vessels, and extracellular matrix components, including sclerosis, whose composition is guided by the neoplastic cells and which in turn, influence tumor initiation, progression, and drug resistance (4). The key factors influencing the composition of microenvironment include lymphoma subtypes and signaling interactions between the lymphoma cells and microenvironment cells. The various components of a typical lymphoma microenvironment are outlined in Table 1.

**Table 1:** Components of lymphoma microenvironment.

A.	IMMUNE CELLLS 1. Cytotoxic T cells (CTLs) 2. Follicular B helper T cells (T_FH_) 3. Regulatory T cells (Tregs) 4. Natural Killer cells (NK) 5. Bystander B cells
B.	STROMAL CELLS 1. Mesenchymal stromal cells (MSC) 2. Lymphoma associated macrophages (LAMs) 3. Myeloid-derived suppressor cells (MDSCs) 4. Dendritic cells
C.	ANGIOGENESIS
D.	EXTRACELLULAR COMPONENTS 1. Extracellular matrix (ECM) 2. Cytokines/Chemokines 3. Lymphoma exosome

A deeper knowledge of interactions between lymphoma cells and its non-malignant microenvironment would be critical in understanding the differences between the pathogenesis and prognosis of various lymphoma subtypes and potential new therapeutic targets.

## Mesenchymal stromal cells (MSCs)

MSCs have both anti-inflammatory as well as immunosuppressive properties. The latter characteristic can aid tumor cells to escape immune surveillance. Investigators have found that co-injection of MSCs with neoplastic (A20) B cells promotes B cell lymphoma growth in the lacrimal glands of immunocompetent mice and were associated with marked increased in CD4+ forkhead box P3 (FoxP3) + T cells and myeloid-derived suppressor cells (5). In murine model of lacrimal gland B-cell lymphomas, those lymphoma cells that were coinjected with MSCs were found to have increased CD4+ Foxp3+ regulatory T cells as well as CD11b+ Ly6C+Ly6G– MDSCs. These coinjected tumors demonstrated less apoptosis and had up-regulated immune-associated molecules such as tumor necrosis factor alpha (TNF-α), interleukin (IL)-1β, transforming growth factor beta (TGF-β), and arginase. Hence, it appears that MSCs help create an immunosuppressive milieu in the context of lacrimal gland B-cell lymphomas (5). Likewise, other investigators have found that MSCs promote tumor growth in mice with p53 mutations that develop spontaneous lymphomas (6).

MSCs have also been postulated to differentiate into the fibroblastic reticular cells and follicular dendritic cells necessary for the infiltration of follicular lymphoma in the bone marrow (7). Investigators have demonstrated that marrow MSCs from patients with follicular lymphoma, which has a relatively high rate of marrow involvement, overexpress chemokine (C-C motif) ligand 2 (CCL2) and aid in sustaining the growth of malignant B cells. These findings suggest an integral role of stromal cells in the infiltration and persistence of lymphoma in medullary sites (7).

## Lymphoma-associated macrophages (LAMs)

LAMs are the macrophage/circulating monocyte lineage cells found in close association with lymphoma. Their roles appear to differ based on tumor type. Elevated numbers of LAMs have been correlated with poor prognosis in certain tumors. In individual studies of advanced stage classic Hodgkin lymphoma (CHL) as well as in meta-analyses, a high-density of LAMs is a strong predictor of adverse outcomes in adult patients (8, 9).

LAMs appear to demonstrate dual predictive roles in follicular lymphoma. High levels of CD68+ or CD163+ LAMs are associated with poor outcome in follicular lymphoma treated with conventional chemotherapy prior to the rituximab era, whereas this effect was diminished or even inverted when rituximab is used in combination (10). In murine models, anti-CD20 monoclonal antibody (mAb) mediated depletion of B cells relied upon the macrophage expression of Fc-gamma receptors (FcγR) (11).

Therapeutically, it has also been shown that relatively novel immunomodulatory drugs such as pomalidomide convert the polarization status of macrophages from M2 to M1 in mouse models of central nervous system (CNS) lymphoma (12). This appears to be achieved by reducing signal transducer and activator of transcription (STAT) 6 signaling while enhancing STAT1 signaling and thereby pomalidomide increases the phagocytic activity of macrophages. This finding argued for the therapeutic activity of pomalidomide against CNS lymphomas.

In CHL, an increased number of CD68 positive LAMs have been significantly associated with a shorter progression-free survival, increased likelihood of relapse after stem cell transplantation and an overall shortened disease-specific survival, making them a potential risk stratification biomarker (13). Such studies were carried out in patients treated with standard chemotherapy so it is unknown whether they would be consistent in patients receiving novel therapies that may alter the microenvironment.

## Myeloid-derived suppressor cells (MDSCs)

MDSCs are myeloid lineage cells that appear to suppress immune surveillance, particularly in the bone marrow. They can accumulate in the context of a wide variety of pathologic conditions, including cancer, and inflammation (14). MDSCs have been shown to form mature osteoclasts in response to nuclear factor KB ligand (RANKL), increasing bone resorption. They are thought to influence the ability of tumors to spread into the marrow niche (15). Tumors can encourage the accrual of MDSCs by secreting factors such as granulocyte-macrophage colony stimulating factor (GM-CSF), stem cell factor (SCF), and interferon-γ (IFN-γ) (16). Elevated levels of MDSCs have been demonstrated in lymphoma, leukemia and multiple myeloma (17).

When normal peripheral blood mononuclear cells were incubated with monocytes from patients with B-cell non-Hodgkin lymphoma (NHL), a reduction in T-cell proliferation as well as decreased Th1-response was seen via measurement of IFN-γ production. Using anti-CD14 immunomagnetic beads to decrease the monocyte population resulted in restored T-cell proliferation. These findings could not be attributed to any significant difference in percentage of monocytes in the peripheral blood of patients vs. healthy controls. Furthermore, the CD14 positive monocytes in patients with NHL showed reduced HLA-DR expression, which is associated with decreased immune function and possibly more aggressive lymphoma (18).

In CHL patients, a group of investigators showed that at initial diagnosis, all subsets of MDSCs were higher in the lymphoma patients compared to healthy controls. While the patients underwent therapy, MDSC subsets declined. The patients who achieved complete response had lower CD34+ MDSCs, monocytic MDSC, and polymorphonuclear MDSCs in their peripheral blood compared to the non-responders. In particular, the undifferentiated CD34+ MDSCs were proposed as a possible biomarker for outcome (19).

More recently, patients with diffuse large B-cell lymphoma (DLBCL) were found to have higher circulating CD14+ HLA-DR^lo^ monocytic MDSCs, which was in concordance with two other studies. The level of these MDSCs correlated with a worse clinical prognosis and was associated with regulatory T cells (Tregs) proliferation (20). Such findings suggest that MDSCs may be a rational target for novel therapies in patients with aggressive lymphomas.

Studies of MDSCs in peripheral T-cell lymphomas and NK/T-cell lymphomas are few and understandably limited in the number of primary human tumors tested. One of the larger studies was conducted in 32 extranodal NK/T cell lymphoma patients. Similar to that found in other lymphoma subtypes, patients with the tumor had higher levels of CD33+ CD11b+ HLA-DR– MDSCs. These MDSCs had increased expression of IL-17, arginase-1 and cytokine-inducible nitric oxide synthase (iNOS) and suppressed T cell proliferation. The higher levels of MDSCs were associated with shorter progression-free survival and overall survival (21). In cutaneous T-cell lymphomas, programmed death-ligand 1 (PD-L1) was expressed by MDSCs as well as by tumor cells themselves and was associated with inhibition of T-cell proliferation and promotion of regulatory FoxP3+ T cells (22).

## Dendritic cells

Dendritic cells are some of the most powerful antigen-presenting cells in the body, aiding in the activation of cytotoxic T cells as well as naïve helper T cells. It has been shown that direct follicular dendritic cell contact with the neoplastic cells of mantle cell lymphomas and other NHL can protect them from apoptosis. This was mediated by upregulation of microRNA-181a (miR-181a), which reduced the levels of proapoptotic Bcl-2-like protein 11 (Bim). Inhibition of miR-181a led to restoration of Bim, releasing the dendritic cell suppression of apoptosis in lymphoma cell lines and primary lymphoma cells (23).

In the setting of follicular lymphoma, tumors with gene expression signatures that included genes highly expressed by dendritic cells and monocytes were associated with poor outcomes. In contrast, those tumors with gene expression signatures containing genes encoding T cell markers and macrophages were associated with prolonged survival (15). However, follow-up studies did not show compatible findings when immunohistochemical assays substituted gene expression analysis (24, 25).

*In vitro* studies were initially promising when DCs were pulsed with either tumor antigen or whole tumor lysate to stimulate immune responses from T cells. While *in vivo* translation into hematologic malignancies have not demonstrated durable responses, these studies were performed in patients with advanced disease (26). Hence, it is possible that combination with other immunotherapy in less advanced disease may be promising.

## Chemokines and cytokines

The microenvironment of CHL is a good model to study the role of chemokines and chemokine receptors in the interaction between microenvironment cells and the Hodgkin Reed-Sternberg (H-RS) cells toward the formation and sustenance of lymphoma microenvironment. The tumor microenvironment of CHL (constituting 99% of the tumor) is composed of B cells, T cells, eosinophils, plasma cells, neutrophils, macrophages, dendritic cells, and fibroblasts, and is largely derived from the dysregulated chemokine secretion by the H-RS cells and TME cells (27). The key cytokines playing an active role in the process, include IL-7, IL-10, TGF-β, chemokine ligand 5 (CCL 5), chemokine ligand 1 (CCL1), and Galectin-1 (28, 29).

The T cells surrounding Reed-Sternberg cells express CCL5, which acts as a chemo-attractant for monocytes, eosinophils, basophils and mast cells as well as CD4 positive T cells (30, 31). C-C chemokine receptor type 3 (CCR3) + Th2 cells and eosinophils are attracted by the CCL1(eotaxin) produced by fibroblasts surrounding RS cells (32, 33). Earlier on, chemokine receptors like C-C chemokine receptor type 5 (CCR5) were thought to be only expressed by the non-neoplastic bystander cells. However, subsequent studies have shown constitutive expression of CCL5 receptor (CCR5) on H-RS cells by immunohistochemistry, flow cytometry, and western blot (34). CCL5, along with other chemokines released by either H-RS cell, Hodgkin cell stimulated fibroblasts or T cells are central to the recruitment of CD4+ T lymphocytes and eosinophils into the classic HL microenvironment. Chronic inflammation at the site of tumor, driven by chemokines and cytokines, has also been found to promote tumor progression (35).

## Cytotoxic T cells (CTLs)

Increased numbers of infiltrating CD8 positive T cells, many expressing cytotoxic markers like TIA-1, as measured by both immunohistochemistry and flow cytometric analysis have been associated with better outcomes in B-cell lymphomas (36, 37). Elevated numbers of cytotoxic lymphocytes positive for programmed cell death-1 (PD-1) was also found to be associated with favorable prognosis in the setting of follicular lymphoma (38).

The cytotoxic activity of T cells is enhanced by the targeting of the PD-1 pathway, which can lead to tumor cell lysis. Tumor specific activated T cells as well as regulatory T cells express cytotoxic T lymphocyte-associated antigen 4 (CTLA-4), which binds to CD80/CD86 on antigen presenting cells and leads to T cell anergy by competing with CD28 as a costimulatory molecule. Immune checkpoint blockade can augment antitumor immunity (39).

During chronic antigen stimulation, a protein called lymphocyte activation gene-3 (LAG-3) is upregulated on T cells, suppressing CD4+ T cell expansion in response to antigen as well as CD8+ T cell function (40). Specifically, LAG-3 has been shown to maintain tolerance to tumor antigens via its effects on CD8+ T cells. In murine models, LAG-3 blockade increases proliferation and effector function of antigen-specific CD8+ T cells within organs and tumors that express their cognate antigen (41). These models suggest that LAG-3 can be a target for increasing the effectiveness of cytotoxic T-cell immunity against tumor.

## Regulatory T cells (Tregs)

Tregs include subsets of immune suppressive cells that regulate self-tolerance and immune homeostasis. Thymic derived Tregs are involved in preventing autoimmunity while peripheral Tregs maintain tolerance in mucosal sites. Both these naturally occurring CD25^+^CD4^+^ Treg populations express FoxP3, which is a more specific marker for regulatory T cells than CD25, CD45RB, or CTLA-4 (41–43). Tregs suppress the activity of bystander T cells, natural killer cells and B cells via CTLA-4, IL-10, and TGF-β1 (44).

FoxP3+ Tregs, particularly in inflamed tissues, have been shown to express T cell immunoglobulin and mucin-domain containing-3 (TIM-3), which enhances their regulatory function. Blockade of TIM-3 signaling appears to demonstrate therapeutic benefit in preclinical tumor models (45). TIM-3 works as a co-inhibitory receptor that is also expressed on IFN-γ producing T cells as well as macrophages and dendritic cells, where it leads to inhibition of normal Th1 responses (46).

Studies in mice have shown that Tregs are present in the peripheral blood of animals and that these circulating cells can regulate humoral immune responses *in vivo*. Furthermore, it was shown that the PD-1 pathway can inhibit blood Treg function. Hence, there is reason to believe that the PD-1: PD-L1 pathway can limit the differentiation and normal function of Tregs, suggesting that manipulation of this pathway can support protective immunity (47).

On the basis of their role in lymphomagenesis, Wang et al divided Tregs into 4 groups: suppressor Tregs (suppress CD8+ CTLs), malignant FoxP3+ Tregs, direct tumor-killing Tregs, and incompetent Tregs. The association between number of Tregs and lymphoma prognosis would vary depending on the type of Tregs present. For instance, in angioimmunoblastic T-cell lymphoma, where more of incompetent Tregs or direct tumor-killing Tregs are present, the anti-tumor cytotoxicity is preserved and hence, better prognosis is associated with increase in Tregs (48).

In certain NHL where Tregs are overrepresented in biopsy specimens compared to normal lymphoid tissue; these cells appeared to be recruited by malignant B cells (49). However, the story is not straightforward. In a study of 280 CHL patients, higher numbers of intratumoral Tregs was associated with better failure free survival and also somewhat better overall survival. Similarly, in follicular lymphoma and germinal center subtype diffuse large B-cell lymphomas, there was a positive correlation between disease specific survival and numbers of intratumoral FoxP3 positive cells (50, 51). From these studies, it has been surmised that the increased Tregs contribute to immune surveillance in lymphomas by reducing overall inflammation and lymphoma cell proliferation.

## Follicular B helper T cells (T_FH_)

T_FH_ cells are abundant in follicular lymphomas. In the normal germinal center, T_FH_ cells appear to be involved in CD40-mediated interactions in the germinal center. In follicular lymphoma, these cells appear to provide IL-4 stimulation to the B cells and in conjunction with CD40 interactions, aid in the proliferation of neoplastic cells through STAT5 signaling (52). Recent work suggests that circulating CD4+ C-X-C chemokine receptor type 5 (CXCR5)+ T cells serve as the memory compartment of T_FH_ cells (53). CXCR5 is the receptor for chemokine ligand 13 (CXCL13), produced by follicular dendritic cells, that promotes the entry of B cells into germinal center. Hence, the upregulated expression of CXCR5 facilitates contact between the B cells and T cells (54).

In patients with low-grade B-cell lymphomas like follicular lymphoma or marginal zone lymphomas, subsets of circulating T_FH_ cells differ from healthy controls, with reduced C-C chemokine receptor type 6 (CCR6) and increased PD-1 (55). Increased levels of PD-1 receptor have also been found in T cells from chronic lymphocytic leukemia (CLL) patients and were not explained by patient age (56). These are correlated with the overexpression of PD-L1 and PD-L2 by the CLL cells. While both CD4+ and CD8+ T cells are increased, overall there are relatively more CD8+ T cells in patients with CLL. The presence of tumor cells appears to be associated with T cells showing an exhausted phenotype. Specifically, they often express CD160, CD244, PR domain zinc finger protein 1 (BLIMP-1), in addition to PD-1 (57). T_FH_ cells have also been shown to provide support for the follicular lymphoma B cells through IL-4 and CD40 ligand production. However, the exact role of T_FH_ cells in the context of lymphoma is not fully understood. Part of the difficulty rests in the fact that they can elicit various cytokine-mediated functions simultaneously and can, in turn, be influenced by their microenvironment (58).

## Natural killer (NK) cells

NK cells are CD16+ CD56+ cytotoxic lymphocytes of the innate immune system, which induce apoptosis even in the absence of antibodies and major histocompatibility complex. NK cells can recognize tumor antigens via killer-cell immunoglobulin-like receptors (KIRs). KIRs can have inhibitory or activating functions and depends on the intracytoplasmic region of the receptor (59). Studies have shown defective NK cell cytolytic function in CLL (60). In a large 11-year human study, low cytotoxic activity of NK cells was associated with increased cancer risk (61).

Working through dendritic cell maturation, NK cells can prune the adaptive immune response. A subset of NK cells produces IFN-γ, TNF-α, IL-10, and certain chemokines that aid in the differentiation of T cells and dendritic cells (62). In mouse models, IFN-γ and perforin protein knockouts will develop B-cell lymphomas that show suggestion of immunosurveillance defect (63). Once a tumor microenvironment is developed, TGF-β is induced and TIM-3 expression on NK cells is upregulated. The increased TIM-3 expression has been associated with lower NK-cell cytotoxicity and poor outcomes in a variety of neoplasms (64).

Studies have demonstrated an acquired quantitative as well as qualitative deficiency of NK cells in CHL microenvironment, contributing to immune evasion mechanism for lymphoma progression (65). A study quantifying immune cells in CHL found NK cell density to be five times less compared to NHL or normal tissues (66). Recent studies have shown significant reduction in NKG2D expression as well as weak cytotoxic activity in NK cells in untreated CHL patients (67). Reactivation of silenced NK cells in CHL is a potential therapeutic target and is being currently pursued. Immune checkpoint inhibitors, like Nivolumab, are being used to recover cytotoxic activity of NK cells in CHL by PD-1 inhibition. Drugs targeting heat shock protein-90 have been found to be effective in preclinical studies (68). In a recent phase 1 study, the bispecific (CD30/CD16a), tetravalent antibody, AFM13 has proven significantly effective in NK cell activation (69).

## Bystander B cells

Bystander CD 20+ B cells are more numerous in lymphocyte predominant Hodgkin Lymphoma (LP-HL) compared with CHL, where their role in tumor progression is debatable (70). B cell production of IL-10 may aid in antitumor immunosuppression by T cell inhibition (71), whereas competition with tumor cells (H-RS) for T-cell derived survival signals may halt tumor cell growth.

## Lymphoma exosome

Exosomes are microparticles that can be secreted by cells and usually range in size from 30 to 100 nm (72). Upon discovery in 1983, they were thought to be cellular waste, but are now known as carriers of signaling molecules in various contexts, ranging from malignant to autoimmune (73) and infectious states (74). They are composed of a bilayer lipid membrane and the internal contents associated with reverse invagination from the plasma membrane and can include mRNAs, microRNAs, proteins, lipids, and signaling molecules (75).

Studies have begun to elucidate the role of exosomes in the interaction between circulating tumor cells and the microenvironment. CLL-derived exosomes were shown to induce stromal cells to take on a cancer-associated fibroblast (CAF) phenotype *in vitro*. The CAFs, in turn, support a niche that promotes CLL cell adhesion, survival and growth *in vivo* (76).

Recent studies demonstrate the possibility of studying circulating lymphoma exosomes. A group from Spain demonstrated the prognostic value of tumor associated mRNA in exosomes of patients with B-cell NHL by utilizing liquid biopsies (77). In this study, BCL-6 and C-MYC positivity in the pretreatment samples predicted worse progression free survival compared to patients without.

In another recent study, exosomes produced by lymphoma B cells carrying mutated *MYD88* were reported to reprogram the marrow microenvironment such that mast cells and macrophages were induced to promote endogenous proinflammatory signaling pathways. Hence, it is believed that exosomes play a key role in the communication of tumor cells to non-malignant cells in the bone marrow, possibly creating a tumor-friendly environment (78).

## Extracellular matrix (ECM)

The extracellular matrix is a network of physically and biochemically distinct macromolecules, like proteins, glycoproteins, and proteoglycans, which constitute the basement membrane and interstitial matrix and are central to the maintenance of structural integrity and regulation of cell behavior in organs (79). In solid organ tumors, dysregulated ECM has been shown to expedite cancer progression directly by affecting cancer cells causing cellular transformation, cancer stem cell expansion and disruption of tissue polarity leading to tumor invasion and metastasis (80) or indirectly by affecting stromal cells (81) and facilitating creation of tumorigenic microenvironment by promotion of angiogenesis and inflammation (82).

## Angiogenesis

Lymphoma tumor microenvironment also includes a rich scaffold of vessels that supply nutrients to the proliferating cells. Much of the prior clinical studies have focused on vascular endothelial growth factor (VEGF) inhibition (83, 84) in preventing tumor angiogenesis. However, the addition of bevacizumab does not currently appear to improve efficacy above that found in R-CHOP chemotherapy alone in the setting of aggressive B cell lymphomas (85).

Platelet-derived growth factor (PDGF) type BB recruits PDGF receptor-expressing pericytes to neovessels, thus promoting vascular maturation and stabilization (86). It appears that PDGF can also be involved in the expression of other stromal angiogenic factors like basic fibroblast growth factor and VEGF (87).

Inhibition of platelet derived growth factor receptor B (PDGFRB) with imatinib mesylate or sunitinib malate has shown some efficacy in carcinoma models (88–90) but has not yet been thoroughly evaluated in the context of lymphomas. One study showed impaired growth of lymphoma in both human xenograft and mouse allograft models with the use of imatinib, a tyrosine kinase inhibitor of PDGFRB. These investigators show decreased microvascular density and *in vivo*, imatinib induced apoptosis of tumor associated PDGFRB positive pericytes and loss of perivascular integrity (91).

The tumor endothelium has also been shown to prevent T cell homing, and hence, can serve as a barrier against immunotherapy. Lessons can be learned and possibly refined from studies carried out in solid organ tumors, such as ovarian cancers, in which overexpression of endothelin B receptor was associated with absence of tumor infiltrating lymphocytes (TILs) and short survival time. An inhibitor for endothelin B receptor increased the adhesion of T cells *in vitro* to human endothelium. This adhesion required intercellular adhesion molecule 1 (ICAM-1) and augmented tumor immunotherapy *in vivo* without increasing systemic antitumor immune response (92). Endothelial mechanisms that regulate how much and which types of T cells can infiltrate the tumor likely plays a large role in the effectiveness of immunotherapy such as cancer vaccines. This area requires much further study, particularly in the setting of lymphomas.

In a study of lymph nodes in 286 Hodgkin lymphoma patients, morphometric parameters of angiogenesis were shown to be related to poor prognosis. Morphometric microvascular parameters, like microvessel density and total vascular area were inversely related to overall disease-specific survival (93).

## Mechanisms of tumor microenvironment mediated immune evasion and tumor progression in NHL

**Figure 1:**
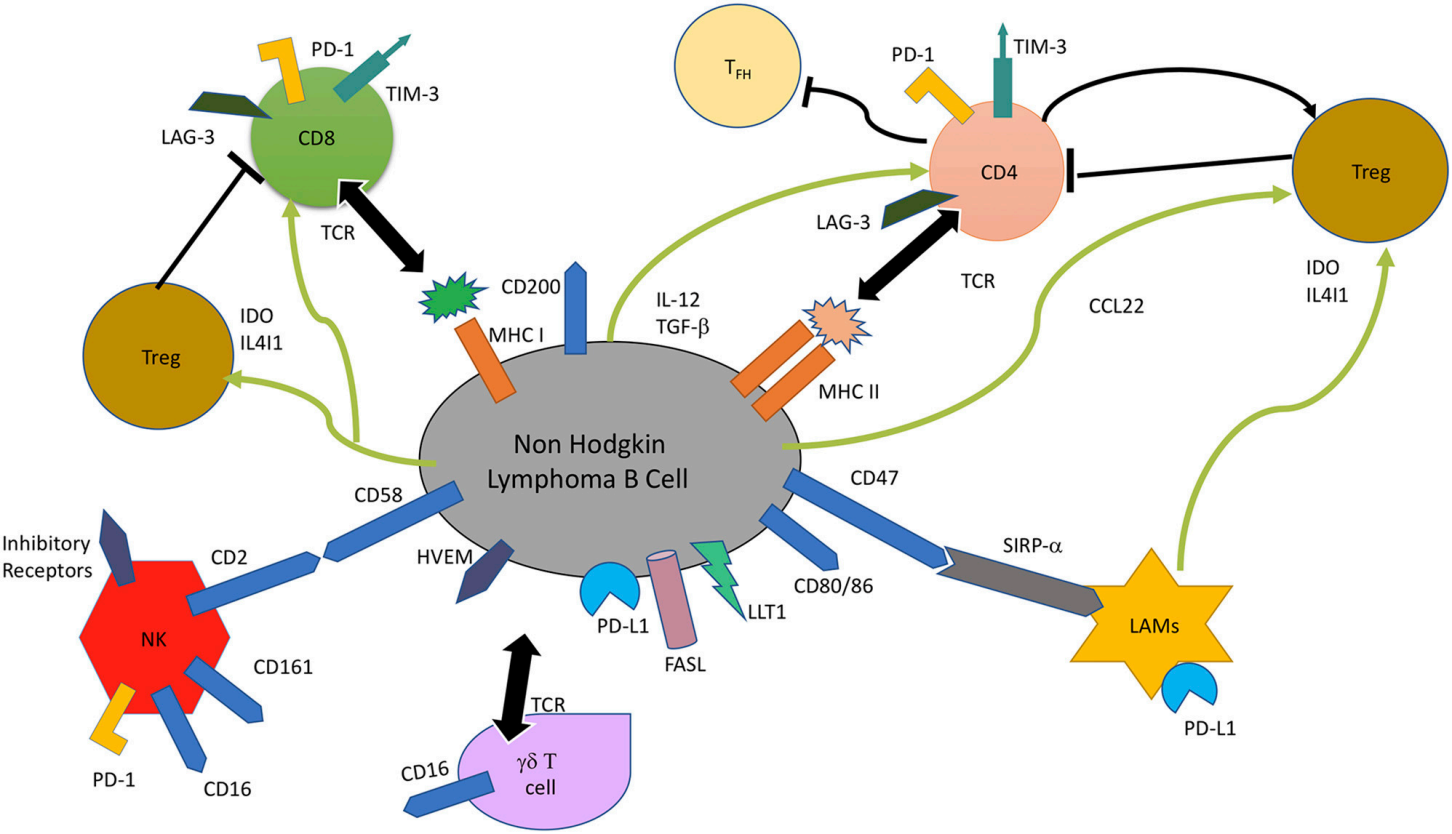
In non-Hodgkin B-cell lymphoma, malignant B cells escape immune response by multiple mechanisms, including lack of recognition by immune cells due to loss of cell surface molecules involved in recognition, CD4+ T cells (MHC II), CD8+ T cells (MHC I), and NK cells (CD58). Overexpression of B cell surface inhibitory receptors, like PD-L1, Lectin-like Transcript 1 (LLT1), Herpes Virus Entry Mediator (HVEM), CD47, and CD200 which are the ligands for PD-1, CD161, BTLA, and SIRP-α, and secretion of inhibitory enzymes, Indoleamine 2, 3-dioxygenase (IDO) and IL4I1 leads to impaired T cell mediated cytotoxicity and T cell exhaustion. IDO and IL4I1 are also responsible for recruitment and differentiation of immunosuppressive Tregs, as well as exhaustion of T-effector cells through CCL22, TGF-β, and IL-12 secretion. FAS Ligand (FASL) induces apoptosis of CTLs (94, 95).

The chief interactions involved in immune escape and promotion of tumor progression in NHL are illustrated in Figure 1. Cytotoxic T lymphocytes (CTLs), gamma delta T (γδ T) cells, natural killer (NK) cells and lymphoma associated macrophages constitute the principal antitumor immune responses in the body. The malignant lymphoma B- cells interact closely in association with the niche microenvironment elements to escape these immune responses.

Loss of lymphoma cell surface molecules/ markers, which are integral to their recognition by immune cells, leads to reduced tumor immunogenicity and immune evasion. Genetic alterations leading to loss of MHC Class I, MHC Class II, and CD58 contribute to the failure of CD 8+ T lymphocyte, CD4+ lymphocyte, and NK cell-mediated tumor cytotoxicity (52). Another mechanism of escaping T/NK cell mediated cytotoxicity is by overexpression of inhibitory lymphoma cell surface molecules, like PD-L1 and herpes virus entry mediator (HVEM), which on interaction with their counterparts on T cells lead to impaired T/NK cell activity (96). It has been shown that the use of anti CD47 antibodies lead to increased phagocytic activity of SIRP-alpha (SIRP-α) bearing macrophages (97), thereby indicating that overexpression of CD47 and SIRP-alpha is a lymphoma cell mechanism to evade macrophage-mediated destruction. The B-NHL cells also modulate the composition of microenvironment toward creation of a more immunosuppressive niche by secretion of Treg chemokine CCL22, in response to IL-4 and CD40L expression by T follicular helper cells (98). Inhibitory enzymes, like indolediamine oxidase (IDO), and phenylalanine oxidase interleukin 4-induced gene 1 (IL4I1), secreted by lymphoma associated macrophages and some B-NHL cells also contributes to immune suppression by Treg expansion and inhibition of effector T cell proliferation and activity (94, 95). Increased expression of FAS Ligand (FASL) by NHL B cell induces cytotoxic T cell apoptosis, whereas IL-12 secretion induces T cell exhaustion by LAG-3 and TIM-3 induction (99).

## Mechanisms of tumor microenvironment mediated immune evasion and tumor progression in CHL

**Figure 2:**
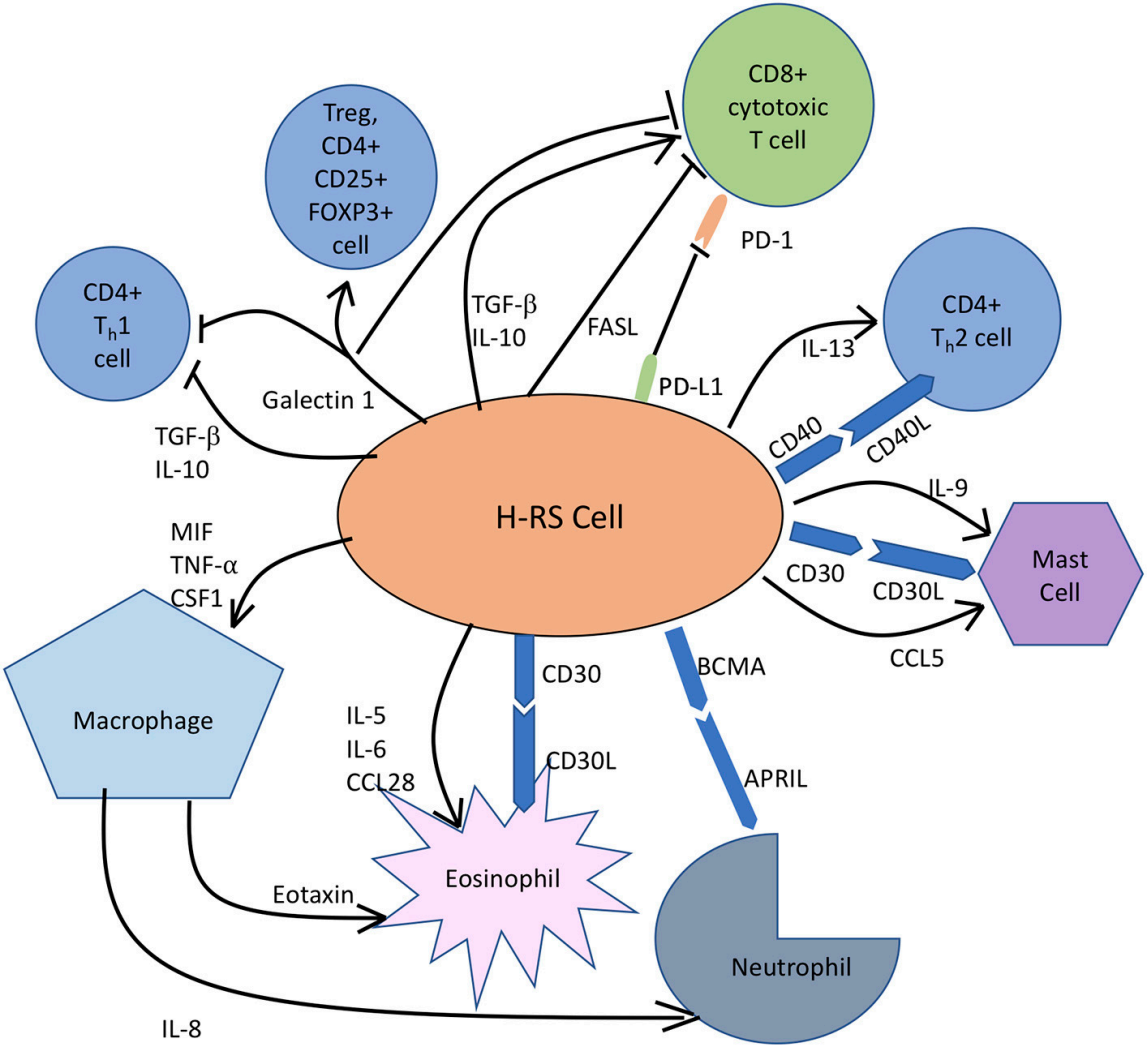
In classic Hodgkin lymphoma, H-RS cells secrete cytokines, like IL-5, IL-9, and IL-10 to recruit eosinophils, mast cells and T cells, respectively to constitute the rich supportive tumor microenvironment. H-RS cells also produce macrophage migration inhibitory factor (MIF), which supports M2 macrophage infiltration. Galectin-1 induces differentiation of CD4+T cells toward immunosuppressive Tregs and causes apoptosis of both T_H_1 cells and CTLs. FAS Ligand (FASL) induces apoptosis of CTLs. PD-L1 expression by H-RS cells helps the tumor escape immune responses by causing T-cell exhaustion. BCMA, B Cell Maturation Antigen; APRIL, Proliferation Inducing Ligand.

The chief interactions involved in immune escape mechanism and promotion of tumor progression in CHL are illustrated in Figure 2. The H-RS cell orchestrates the rich polymorphous background cellularity comprising of T cells, macrophages, eosinophils, mast cells, neutrophils, plasma cells, plasma cells, stromal cells, and fibroblasts principally through secretion of cytokines and chemokines. H-RS cells secrete Colony Stimulating Factor-1 (CSF-1) and macrophage migration inhibitory factor (MIF) to recruit M2 macrophages, which in turn, secrete chemokines like, IL-8, to attract neutrophils into and eotaxin to attract eosinophils into tumor tissue (52).

It has been widely appreciated that TME and H-RS cells contribute to anti-tumor immune evasion by multiple mechanisms. Loss of MHC Class II molecules in CHL by chromosomal translocation has been linked to reduced tumor antigen presentation and hence, escape from immune cells. Aberrant expression by H-RS cells of surface molecule PD-L1, the ligand for PD- expressed on CTLs and CD4+ T cells, reduces anti-tumor immune function by T cell exhaustion. H-RS cells modify the microenvironment composition toward an immune tolerant state by inducing CD4+ T cell differentiation into immunosuppressive Tregs by secreting Galectin-1, TGF-β and CD70 and CD80 expression or by causing T-cell exhaustion through the secretion of TGF-β, IL-10, galectin-1, and prostaglandin E2 (100). Expression of FAS Ligand can induce apoptosis of CTLs, leading to reduced T cell mediated tumor cytotoxicity and tumor progression (101).

## Therapeutic implications

A better understanding of the interactions between the lymphoma cells and the microenvironment niche has unraveled multiple new potential therapeutic targets in lymphoma treatment. The use of active and passive immunotherapy to bolster antitumor response is one such strategy and has been found to be considerably successful (102). Passive immunotherapy, based on the use of monoclonal antibodies and genetically engineered T cells has shown promising results in the treatment of relapsed/refractory NHL (103, 104). Recently, newer antibodies with multiple binding sites for tumor and T cells are being developed and early clinical trial results using bispecific T-cell engager (BiTE), blinatumomab have been very promising (104).

Active immunotherapy modalities include vaccines and immune checkpoint inhibitors. The results with vaccines have been variable. Immune checkpoint inhibitors, on the other hand, have yielded excellent response rates, especially in Hodgkin lymphoma (60–80%) compared to NHL (20–40%) (105).

Improving the function of infiltrating immune effector cells, like T cells, and macrophages, has been shown to improve survival. Another major focus of upcoming lymphoma treatment strategies has been to target and diminish the microenvironment support for tumor cells, thereby limiting their survival. These treatment modalities have included targeting the pro-survival cell surface molecule signaling pathways (protumor signals), limiting tumor angiogenesis, attacking protumor microenvironment cells like mesenchymal stromal cells.

Similar to disrupting the protumor microenvironment approach is the recent focus on therapeutics aimed at mobilization of lymphoma cells away from their nourishing microenvironment. Abnormal ECM architecture, like dense collagen, has been known to be associated with poor chemotherapy response and resistance in solid tumors due to impaired drug delivery (106). In murine models, vaccine targeting tumor associated fibroblasts has been proven to decrease collagen type I expression, leading to 70% greater drug uptake (107).

## Conclusion

The lymphoma microenvironment is a complex stage where the actors can interact with each other in varying ways depending on the context. It is becoming clear that the so-called bystander cells of the microenvironment may share the limelight with tumor cells in their contribution to disease pathogenesis and progression. Understanding their function can lead to more sophisticated methods of turning host cells effectively against the lymphoma as well as to circumvent resistance against immune checkpoint blockade and life-threatening complications from therapy.

## Author contributions

DK designed, researched, wrote and revised the manuscript. MX conceived, designed, researched, wrote and edited this paper.

### Conflict of interest statement

The authors declare that the research was conducted in the absence of any commercial or financial relationships that could be construed as a potential conflict of interest.
