# Corrigendum: Microenvironment Cell Contribution to Lymphoma Immunity

**DOI:** 10.3389/fonc.2018.00522

**Published:** 2018-11-08

**Authors:** Deepika Kumar, Mina L. Xu

**Affiliations:** Departments of Pathology & Laboratory Medicine, Yale University School of Medicine, New Haven, CT, United States

**Keywords:** lymphoma, microenvironment, T cell subsets, stromal cells, lymphoma exosomes

In the original article, Boulland et al. (94) and Carbonelle-Puscian et al. (95) were not cited in the article. These references have now been inserted in Mechanisms of Tumor Microenvironment Mediated Immune Evasion and Tumor Progression in NHL, **Paragraph Number 2**:

Inhibitory enzymes, like indolediamine oxidase (IDO), and phenylalanine oxidase interleukin 4-induced gene 1 (IL4I1), secreted by lymphoma associated macrophages and some B-NHL cells also contributes to immune suppression by Treg expansion and inhibition of effector T cell proliferation and activity (94, 95).

The citations for Boulland et al. (94) and Carbonelle-Puscian et al. (95) have also been inserted in the legend for Figure 1:

IDO and IL4I1 are also responsible for recruitment and differentiation of immunosuppressive Tregs, as well as exhaustion of T-effector cells through CCL22, TGF-β, and IL-12 secretion. FAS Ligand (FASL) induces apoptosis of CTLs (94, 95).

The authors apologize for this error and state that this does not change the scientific conclusions of the article in any way. The original article has been updated.

## Conflict of interest statement

The authors declare that the research was conducted in the absence of any commercial or financial relationships that could be construed as a potential conflict of interest.
